# Supplementary material for: A Colorimetric Strategy Based on Aptamer-Catalyzed Hairpin Assembly for the On-Site Detection of Salmonella typhimurium in Milk
Source: Foods. 2021 Oct 22;10(11):2539. doi: 10.3390/foods10112539 (PMC8623332; doi:10.3390/foods10112539)
Supplement: Supplementary file 1 [file foods-10-02539-s001.zip › foods-1427651-supplementary.pdf]

SUPPLEMENTARY MATERIALS

A colorimetric strategy based on aptamer catalyzed hairpin assembly for the on-site detection of *Salmonella typhimurium* in milk

Sihan Chen [1], Xinran Zong [1], Jiapeng Zheng [1], Jiaxin Zhang [1], Mengyao Zhou [1], Qing Chen [1], Chaoxin Man [1,*] and Yujun Jiang [1,*]

[1] Key Laboratory of Dairy Science, Ministry of Education, Department of Food Science, Northeast Agricultural University, Harbin 150030, China

[*]Corresponding authors: yujun_jiang@163.com and mcxwh2006@qq.com

Table S1. The sequences of the oligonucleotides in this work.

Name	Sequence (5' to 3')
Bapt	biotin-GAGGAAAGTCTATAGCAGAGGAGATGTGTGAACCGAGTAA
Tapt	TATGGCGGCGTCACCCGACGGGGACTTGACATTATGACAG
Y1	CTGTCATAATGTCAAGTCCCCGTCGGGTGACGCCGCCATATATGGCGGCGCGCCGCCATATATGGCGGCGTCACCCGACGGGGACTTGAC
Y2	CGTCGGGTGACGCCGCCATATATGGCGGCGCGCCGCCATAGGGACTTGACATTATGACAGTATGGCGGCGCGCCGCCATATATGGCGGCG
Y3	TATGGCGGCGCGCCGCCATACTGTCATAATGTCAAGTCCCTATGGCGGCGTCACCCGACGGGGACTTGACATTATGACAGTATGGCGGCG
Y1*	GTCAAGTCCCCGTCGGGTGACGCCGCCATATATGGCGGCGCGCCGCCATATATGGCGGCGTCACCCGACGGGGACTTGAC
Y2*	CGCCGCCATATATGGCGGCGCGCCGCCATAGGGACTTGACATTATGACAGTATGGCGGCGCGCCGCCATATATGGCGGCG
Y3*	CGCCGCCATACTGTCATAATGTCAAGTCCCTATGGCGGCGTCACCCGACGGGGACTTGACATTATGACAGTATGGCGGCG
C1	CTGTCATAATGTCAAGTCCCCGTCGGGTGACGCCGCCATACTATCTACTTACTCTTACTTTATGGCGGCGTCACCCGACGGGGACTTGAC
C2	CGCCGCCATAAAGTAAGAGTAAGTAGATAGTATGGCGGCGTCACCCGACGGGGACTTGACCTATCTACTTACTCTTACTT

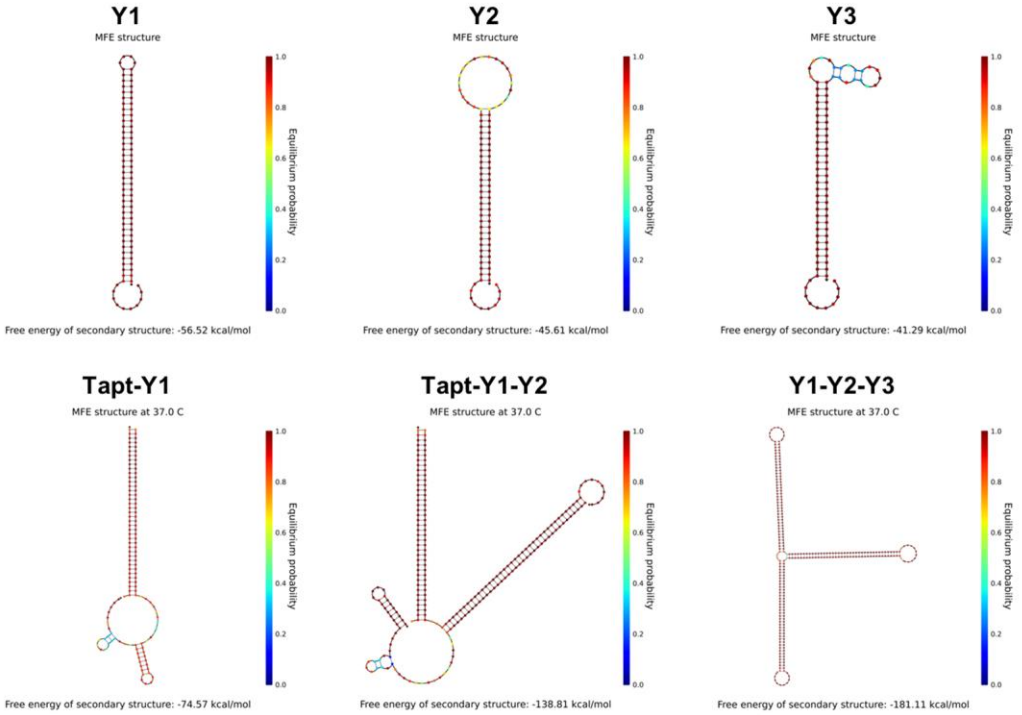

Figure S1. Simulation and prediction on the secondary structures of the hairpins Y1, Y2, Y3 and hybrids based on the NUPACK software.

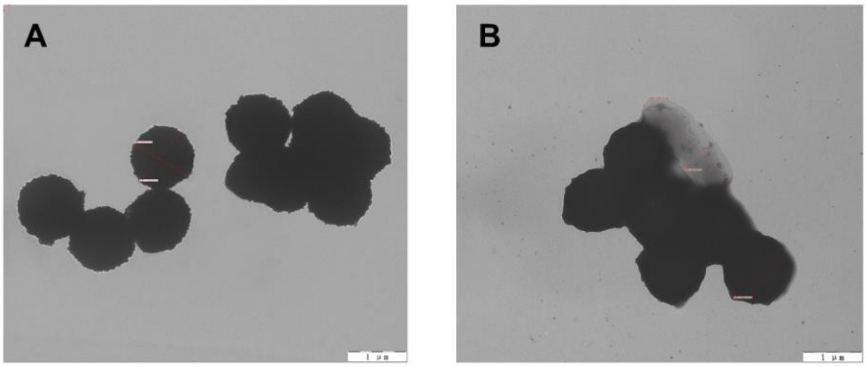

Figure S2. TEM image of SMBs (A) and SMBs-Bapt-*S. typhimurium* (B).

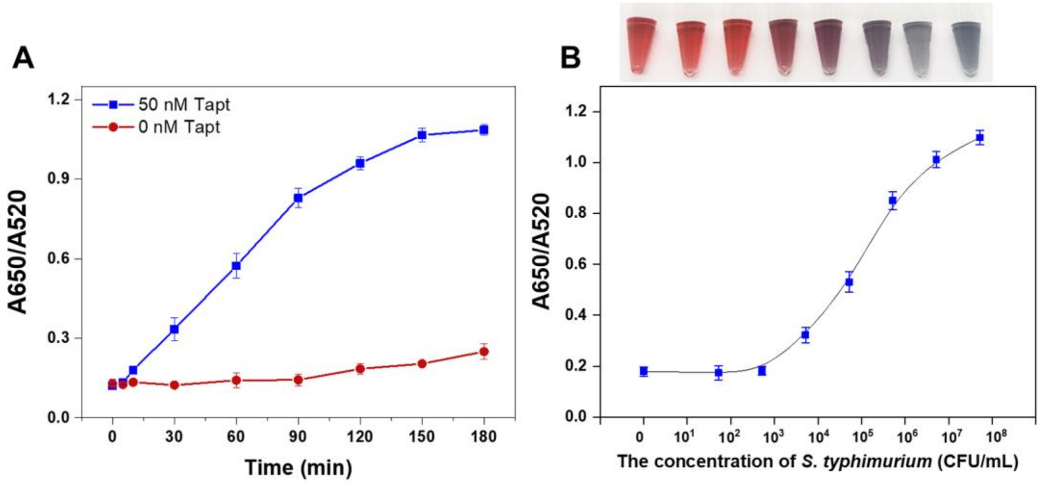

Figure S3. Effect of incubation time on conventional CHA (A). Sensitivity of conventional CHA for detection of *S. typhimurium* with concentrations ranging from 5.2×10^1 to 5.2×10^7 CFU/mL (B). The insert shows the AuNPs visualization colorimetric photo of the corresponding bacterial sample.

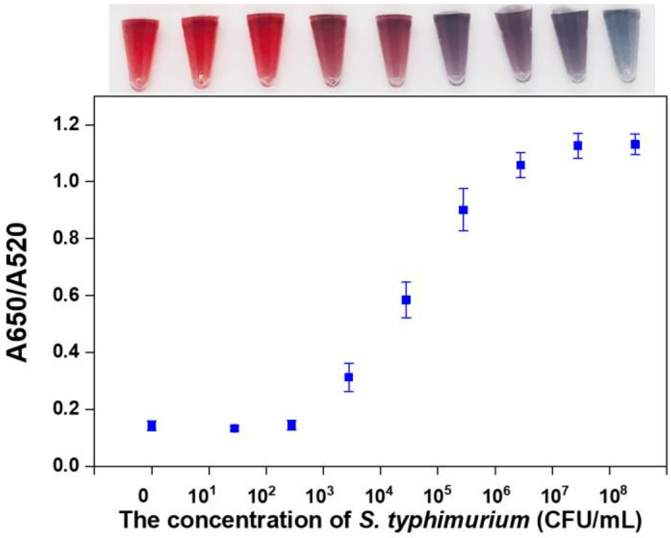

Figure S4. Detection of *S. typhimurium* in contaminated milk using the on-site colorimetric strategy based on Y-CHA circuit. The insert shows the AuNPs visualization colorimetric photo of the corresponding bacterial sample.
